# Anti-obesity Effect of Capsaicin in Mice Fed with High-Fat Diet Is Associated with an Increase in Population of the Gut Bacterium *Akkermansia muciniphila*

**DOI:** 10.3389/fmicb.2017.00272

**Published:** 2017-02-23

**Authors:** Wei Shen, Mengyu Shen, Xia Zhao, Hongbin Zhu, Yuhui Yang, Shuguang Lu, Yinling Tan, Gang Li, Ming Li, Jing Wang, Fuquan Hu, Shuai Le

**Affiliations:** Department of Microbiology, Third Military Medical UniversityChongqing, China

**Keywords:** gut, micriobiome, capsaicin, *Akkermansia*, mucin

## Abstract

Capsaicin (CAP) reduces body weight mainly through activation of transient receptor potential vanilloid 1 (TRPV1) cation channel. However, recent evidence indicates that the gut microbiota influences many physiological processes in host and might provoke obesity. This study determined whether the anti-obesity effect of CAP is related to the changes in gut microbiota. C57BL/6 mice were fed either with high-fat diet (HFD) or HFD with CAP (HFD-CAP) for 9 weeks. We observed a significantly reduced weight gain and improved glucose tolerance in HFD-CAP-fed mice compared with HFD-fed mice. 16S rRNA gene sequencing results showed a decrease of phylum *Proteobacteria* in HFD-CAP-fed mice. In addition, HFD-CAP-fed mice showed a higher abundance of *Akkermansia muciniphila*, a mucin-degrading bacterium with beneficial effects on host metabolism. Further studies found that CAP directly up-regulates the expression of Mucin 2 gene *Muc2* and antimicrobial protein gene *Reg3g* in the intestine. These data suggest that the anti-obesity effect of CAP is associated with a modest modulation of the gut microbiota.

## Introduction

Obesity is a global health problem that increases the risk of many physical and mental conditions, such as metabolic syndrome (Ogden et al., 2007). Diet regulation and exercise are the main treatments to obesity. Several molecules from diet have been shown to reduce body weight. CAP is an ingredient of chili peppers, which are consumed worldwide as vegetables and flavorings (Yang et al., 2010). The anti-obesity effect of CAP has been extensively reported in both human and animal studies (Derbenev and Zsombok, 2016), but the mechanisms are very complicated, which include regulation of whole body metabolism or reduction of food intake. Ludy et al. (2012) reported a modest correlation of CAP consumption and body weight reduction. CAP activates the TRPV1 cation channel and enhances catecholamine secretion from adrenal medulla to increase thermogenesis and to reduce weight gain and adipogenesis (Kawabata et al., 2006; Hachiya et al., 2007). CAP can also enhance thermogenesis by activating gastrointestinal TRPV1 in mice (Kawabata et al., 2009). Lee et al. (2015) found that CAP can reduce food intake, which is dependent on TRPV1. The anti-obesity effect of CAP via TRPV1 activation has been extensively studied, but the impact of CAP on gut microbiota has not been well studied.

Recently, growing evidences showed that dysbiosis of gut microbiota plays a significant role in the development of obesity and metabolic syndromes (Sonnenburg and Backhed, 2016; Wang and Jia, 2016). The gut microbiota has numerous physiological and pathological interactions with the host, such as host energy balance regulation, glucose metabolism, and the chronic inflammatory state (Sonnenburg and Backhed, 2016). Profound changes in the composition of the gut microbiota have been uncovered in obese mice and humans. HFD increased the levels of phyla *Firmicutes* and *Proteobacteria* and decreased the abundance of *Bifidobacterium* spp. and *Lactobacillus gasseri*, which are known to have beneficial effects on hosts (Wang and Jia, 2016).

At the species level, the abundance of *Akkermansia muciniphila* has been shown to inversely correlate with obesity and type I diabetes in both mice and humans (Everard et al., 2013; Shin et al., 2014; Dao et al., 2016). *A. muciniphila* is a mucin-degrading bacterium that can use gastric mucin as the sole carbon and nitrogen source (Derrien et al., 2004, 2011; Collado et al., 2007; Derrien, 2007; van Passel et al., 2011). The oral administration of *A. muciniphila* prevents HFD-induced obesity by altering adipose tissue metabolism and gut barrier function (Everard et al., 2013). The anti-obesity effect of a polyphenol-rich cranberry was associated with increased *Akkermansia* spp. population in the mice gut (Anhê et al., 2015). Therefore, these experiments showed that gut microbiota dysbiosis may stimulate the development of obesity and the modulation of gut microbiome may reduce body weight (Derrien et al., 2016; Gomez-Gallego et al., 2016).

Recently, Baboota et al. (2014) detected a significant gut microbial alteration in CAP treated mice by qPCR, but the detailed changes in microbiome are lacking. In this study, we explored the effects of CAP on gut microbiota in HFD-fed mice by high throughput sequencing and determined whether the anti-obesity effect is related to the modulation of the gut microbiota.

## Materials and Methods

### Animals and Diets

C57BL/6J male mice (Animal Center, Third Military Medical University) were bred, no more than six mice per cage in the animal facility of Third Military Medical University. Animals were housed in a controlled environment (12 h per day/night cycle and lights off at 19:00) with free access to food and water. All animal protocols were approved by the institute of Animal Care and Use Committee (Approval number: SYXC-2014-00203). The animal experiments were performed in accordance with the Guidelines for the Care and Use of Laboratory Animals at Third Military Medical University.

After 1 week of acclimation (week 0) on a normal chow diet, 5-week-old mice (*n* = 12) were randomly divided into two groups of six and fed the following diets (Luo et al., 2012): (1) a HFD containing 45% fat and (2) a HFD with 0.01% CAP (a gift from Prof. Zhiming Zhu, Daping Hospital, Third Military Medical University) treatment in food (HFD-CAP). Animals were given unrestricted access to food and water.

Body weight and average food intake were monitored every day in the 1st week (week 1) after treatment and once a week after week 1. During week 9, mice feces were collected and stored at −80°C until DNA extraction, and oral glucose tolerance test (OGTT) was assessed. At last, animals were anesthetized with amobarbital and then sacrificed by cardiac puncture. Gut tissues were collected for histological staining or tissue RNA extraction.

For RT-qPCR experiment, sixteen 6-week C57BL/6J male mice were randomly divided into four groups and all were fed orally with CAP (0.4 mg in 200 μL PBS and 0.4 mg is computed according to the daily consumption of 4 g of chow per mice multiplied by 0.01%), At the four time points (0, 30, 60, and 90 min), one group of mice (*n* = 4) were anesthetized with amobarbital, and the jejunum and proximal colon tissue samples were collected for RNA extraction.

### Glucose Homeostasis

Mice were fasted overnight (17:00–9:00), and an OGTT was performed after gavage with glucose (1 g/kg body weight, volume: ∼200 μL) (Anhê et al., 2015). Blood glucose concentrations were measured with glucometer (Johnson, USA) before (0 min) and after (15, 30, 60, 90, and 120 min) glucose challenge (Ayala et al., 2010; Anhê et al., 2015).

### Gut Microbiota Analysis

Fecal bacterial DNA was extracted using QIAamp DNA Stool Mini Kit (QIAGEN, German) from approximately 100 mg feces according to the manufacturer’s protocol. For each sample, the V4 hypervariable region of the bacterial 16S rRNA gene was amplified using primers 515F (5′-GTGCCAGCMGC CGCGGTAA-3′) and 806R (5′-GGACTACHVGGGTWTCTAA T-3′) (Caporaso et al., 2011), purified, and then sent to Novogene Corporation for the generation of sequencing libraries by using TruSeq DNA PCR-Free Sample Preparation Kit (Illumina, USA) and sequenced on the Illumina Hiseq 2500 platform.

After de-multiplexing of the paired-end 250 bp raw reads, Trimmomatic (v0.32) (Bolger et al., 2014) was used to clean low-quality reads and remove adapter sequences. Paired ends were merged using FLASH (v1.2.7) (Magoc and Salzberg, 2011) and filtered under specific filtering conditions to obtain high-quality clean tags according to the QIIME (v1.91) (Caporaso et al., 2010) quality control process. The OTU picking processing adopted an open-reference OTU picking protocol by searching reads against the Greengenes (gg_13_8) (DeSantis et al., 2006) database (similarity ≥ 0.97) according the QIIME tutorial^1^ and the diversity analyses followed the same tutorial. LEFSe (Version 1.0) (Segata et al., 2011) was used to discover the metagenomic biomarker for both organisms and pathways. PICURSt (Version 1.0.0-dev) (Langille et al., 2013) and HUManN (Version 0.99) (Abubucker et al., 2012) were used to predict functional profiling of microbial communities using 16S rRNA amplicon sequencing result following the PICURSt tutorial^2^^,^^3^ .

Determination of absolute abundance of *A. muciniphila* followed the method from Everard et al. (2013). Briefly, *A. muciniphila* specific primers (forward: 5′-CAGCACGTGAAG GTGGGGAC-3′, reverse: 5′-CCTTGCGGTTGGCTTCAGAT-3′) were used to detect *A. muciniphila* through real time PCR. A standard curve (performed in triplicate) was made by fivefold serial dilutions of genomic DNA of *A. muciniphila* (ATCC BAA-835). And the cycle threshold of each sample was compared with the standard curve.

### Bacterial Strains and Growth Curve

*Akkermansia muciniphila* strain ATCC BAA-835 was purchased from the ATCC. Strains were cultured in BHI medium in tubes at 37°C in anaerobic chamber (Whitley A35 Workstation 2.5, Don Whitley Scientific, UK). To acquire the growth curve of *A. muciniphila*, 3 mL cell cultures were grown with different concentrations (2, 20, and 200 μg/mL) of CAP or with equal volume (2 μL for 1 mL medium) of ethanol. Mucin (from porcine stomach, Type II, Sigma, USA, #SLBH5886V) was added to the final concentration of 4 g/L when needed. CAP (Sigma, USA, #MKBS2249V) was dissolved in ethanol. Cell density was measured by serial 10-fold dilution and plating on BHI agar plates (1% agar). Each experiment was repeated three times.

### Gene Expression

Total RNA was extracted from the jejunum and proximal colon using TriPure (Roche, Switzerland). Genomic DNA removal and cDNA synthesis were performed using TransScript II One-Step gDNA Removal and cDNA Synthesis SuperMix (Transgen, China). Real-time PCR was performed using SYBR Premix Ex Taq II (Takara, Japan) with 1:5 diluted cDNA products.

Target genes including *Muc2* and *Klf4* (a goblet cell marker), and *Reg3g* (an antimicrobial marker), gene were detected with hypoxanthine guanine phosphoribosyl transferase (*Hprt*) as the housekeeping gene (Anhê et al., 2015). Data were calculated according to the 2^-ΔΔCt^ method.

### Goblet Cell Staining

The distal ileum and proximal colon were stained with PAS, (Sigma, USA) as previously described (Shin et al., 2014). The goblet cells were stained pink to red and nuclei were blue.

### Statistical Analysis

Weight, glycemia, goblet cell count and gene expression level data are expressed as mean ± SEM. For different abundance taxon analyses in STAMP (Parks et al., 2014), Welch’s *t*-test was used for the comparison of two groups. One way ANOVA followed by Newman–Keuls *post hoc* was used for real time PCR results tests. Student’s *t*-test was used for other cases.

## Results

### Anti-obesity Effect of Capsaicin in Mice Fed with High-Fat Diet

**FIGURE 1:**
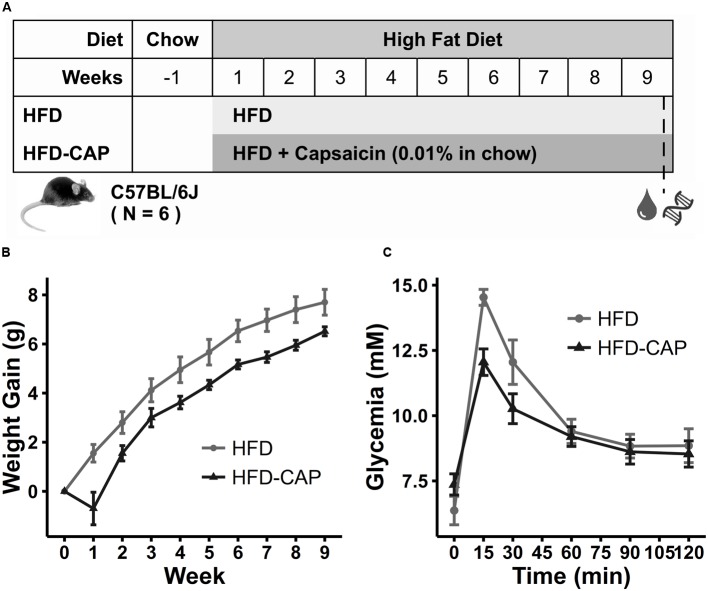
**(A)** Groups information. Five-week-old male mice (*n* = 12) were randomly divided into two groups of six and fed the following diets: (1) a HFD containing 45% fat and (2) a HFD with 0.01% capsaicin treatment in food (HFD-CAP). **(B)** Gained body weight within 9 weeks. **(C)** Oral glucose tolerance test (OGTT) was performed after gavage with glucose (1 g/kg body weight). Blood glucose concentrations were measured with glucometer before (0 min) and after (15, 30, 60, 90, and 120 min) glucose challenge.

To assess the effect of CAP on obesity, 5-week-old male C57BL/6 mice were fed either with HFD or HFD-CAP diet for 9 weeks (**Figure 1A**). The HFD-CAP-fed mice had a lower (*P* < 0.05) body weight gain (6.52 ± 0.45 g) than HFD-fed mice (7.7 ± 1.29 g) (**Figure 1B**; **Supplementary Figure S1A**), which was associated with reduction of food intake (**Supplementary Figure S1B**). The sharp reduction of weight at week 1 could be explained by the reduction of food intake during the early days after suddenly change of food containing spicy CAP. Next, we used a glucose tolerance test to examine glucose homeostasis in CAP-treated and non-treated mice. Compared with HFD-fed mice, HFD-CAP-fed mice showed a smaller (*P* < 0.05) AUC (**Figure 1C**; **Supplementary Figure S1C**), which indicated an improvement in glucose tolerance. These findings confirmed that dietary CAP helped prevent obesity and improved glucose homeostasis under HFD conditions.

### Capsaicin Altered the Gut Microbiota and Increased the Abundance of *Akkermansia*

Fecal DNA of all the 12 mice at week 9 were extracted and sequenced by 16S rRNA gene amplicons sequencing. The average effective tags per sample were 54 thousand (ranging from 47 thousand to 63 thousand tags/sample).

**FIGURE 2:**
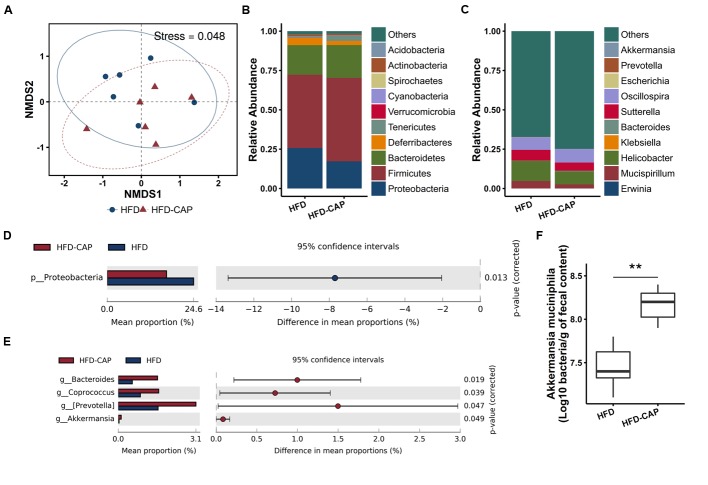
**Microbiome composition. (A)** NMDS for ordination plot of normalized counts at the OTU level (stress = 0.048). **(B,C)** Taxonomy composition at the phylum and genus levels. The top 10 phyla/genera were reported for each group, and all others are grouped into “others.” **(D,E)** Taxa that differ significantly between HFD-CAP and HFD at the phylum and genus level. **(F)** Absolute abundance of *Akkermansia muciniphila* in fecal content by qPCR. ^∗∗^*P* < 0.01.

The NMDS plot of the microbial compositions of the 12 samples revealed that the HFD and HFD-CAP group have similar microbiome composition (**Figure 2A**). However, at the phylum level, among the top 10 abundant phyla, the average proportion of *Acidobacteria*, *Bacteroidetes*, and *Firmicutes* was increased in HFD-CAP group, while the abundance level of *Deferribacteres* and *Proteobacteria* was decreased (**Figure 2B**). According to Welch’s *t*-tests by STAMP (Parks et al., 2014), only the decrease (from 24.63 ± 4.18% to 16.92 ± 3.83%) of *Proteobacteria* abundance is significant (*P* = 0.013) (**Figure 2D**).

At the genus level, among the top 10 abundant genera, the average proportion of *Helicobacter* and *Mucispirillum* was decreased in the HFD-CAP group (**Figure 2C**). According to Welch’s *t*-tests by STAMP, the abundance levels of *Bacteroides* (from 0.56 ± 0.35% to 1.55 ± 0.66%), *Coprococcus* (from 0.88% ± 0.32% to 1.60 ± 0.57%), *Prevotella* (from 1.58 ± 0.84% to 3.07 ± 1.18%), and *Akkermansia* (from 0.03 ± 0.04% to 0.12 ± 0.07%) increased significantly (**Figure 2E**). Real-time PCR also confirmed the increase (*p* < 0.01) of absolute abundance of *A. muciniphila* (**Figure 2F**) in HFD-CAP group.

**FIGURE 3:**
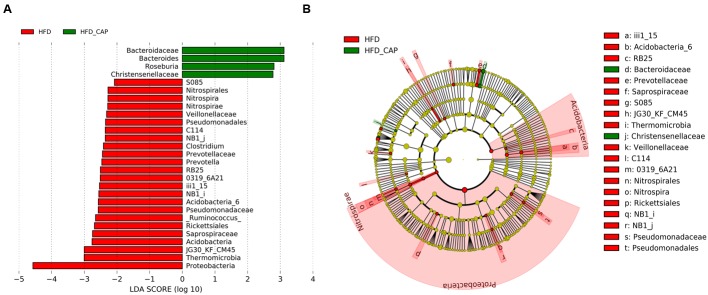
**LEfSe identified the most differentially abundant taxa at the genus level between HFD-CAP and HFD group. (A)** HFD-CAP-enriched taxa are indicated with a positive LDA score (green) and taxa enriched in HFD with a negative score (red). Only taxa meeting an LDA significant threshold of >2 are shown. **(B)** Taxonomic cladogram obtained from LEfSe analysis of 16S sequences HFD-enriched taxa (Red); taxa enriched in HFD-CAP (Green). The brightness of each dot is proportional to its effect size. Each circle’s diameter is proportional to the taxon’s abundance.

We compared the fecal microbiota in HFD and HFD-CAP groups using LEfSe (Segata et al., 2011) to identify the specific bacterial taxa associated with treatment of CAP. A cladogram representative of the structure of the fecal microbiota was shown in **Figure 3B**. The greatest differences in multiple levels of taxa between the two communities were displayed (**Figure 3A**). These data indicated the significantly decreased phylum *Proteobacteria* can be one of the biomarkers of HFD group. Family *Bacteroidaceae* and its genus *Bacteroides* can be the biomarkers of HFD-CAP group.

**FIGURE 4:**
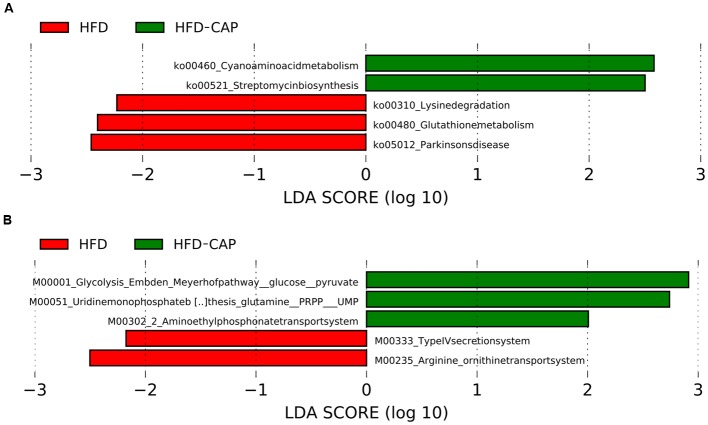
**LEfSe identified the most differentially abundant PICRUSt-predicated KEGG pathway between HFD-CAP and HFD group.** HFD_CAP-enriched pathway **(A)** and module **(B)** are indicated with a positive LDA score (green), and pathway **(A)** and module **(B)** enriched in HFD with a negative score (red). Only taxa meeting an LDA significant threshold of >2 are shown.

The changes in microbial taxa are associated with changes in functional gene abundances, as predicted from 16S rRNA data analysis using the PICRUSt (phylogenetic investigation of communities by reconstruction of unobserved state) software (Langille et al., 2013). LEFSe identified that three KEGG pathways, including lysine degradation, Glutathione metabolism, and Parkinsons disease, were enriched in HFD group, while two pathways, including cyanoamino acid metabolism and streptomycin biosynthesis, are enriched in HFD-CAP group (**Figure 4A**). At the module level (**Figure 4B**), the type IV secretion system modules and arginine/ornithine transport system are enriched in HFD group. Glycolysis (Embden-Meyerhof pathway), uridine monophosphate biosynthesis, and aminoethyl phosphonate transport system are enriched in HFD-CAP group.

### Capsaicin Is Toxic to *A. muciniphila*, But Mucin Promotes *A. muciniphila* Growth *In vitro*

**FIGURE 5:**
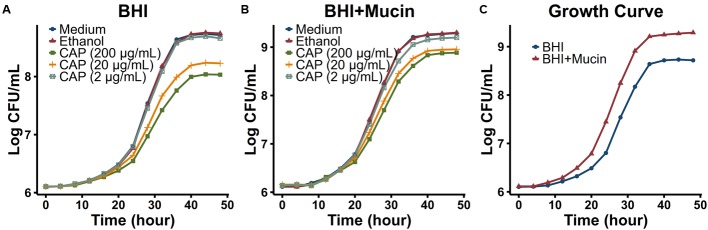
**Growth curves of *Akkermansia muciniphila* in different conditions.** *A. muciniphila* strain ATCC BAA-835 was cultured in BHI **(A)** or BHI+Mucin **(B)** medium with different concentrations (2, 20, and 200 μg/mL) of CAP (dissolved in ethanol) or with equal volume (2 μL for 1 mL medium) of ethanol. **(C)** Growth curves of *A. muciniphila* in BHI and BHI+Mucin medium without CAP or ethanol.

To test whether CAP directly stimulated the growth of *A. muciniphila in vitro*, the growth curve of *A. muciniphila* strain ATCC BAA-835 was monitored in both BHI medium and BHI medium with mucin (from porcine stomach, Type II) (**Figure 5**). High concentration of CAP (20 or 200 μg/mL) inhibited the growth of *A. muciniphila*, whereas low concentration of CAP (2 μg/mL) did not promote the growth of *A. muciniphila in vitro* directly (**Figures 5A,B**). However, the addition of mucin directly promoted the growth of *A. muciniphila* in BHI medium (**Figures 5B,C**). When mucin was provided, *A. muciniphila* grew faster at log phase and plateaued at a much higher cell density. Thus, mucin could promote *A. muciniphila* growth.

### Capsaicin Increases *Muc2* and *Reg3g* Gene Expression in the Intestine

**FIGURE 6:**
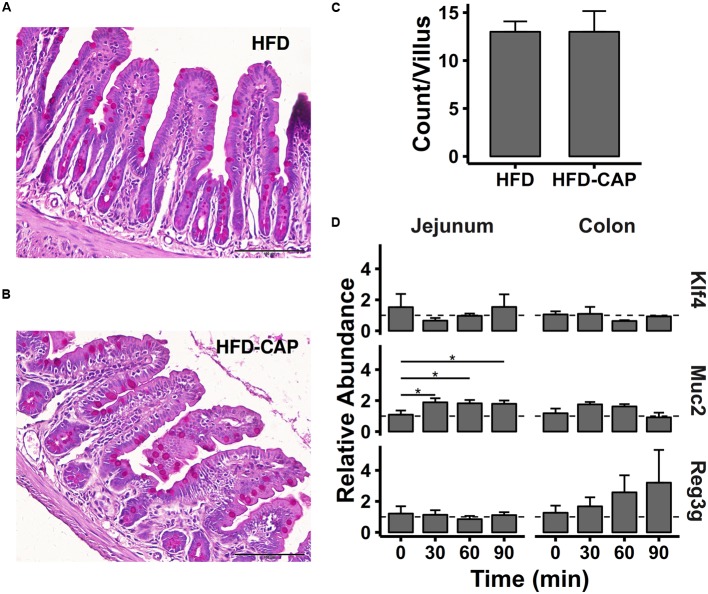
**(A,B)** Representative red photomicrographs showing ileal section stained with periodic acid-Schiff (PAS), and goblet cells were stained pink to red and nuclei were blue. **(C)** Count number of PAS-positive goblet cells per villus. **(D)** Gene expression level of *Klf4*, *Muc2*, and *Reg3g* (*n* = 4). ^∗^*P* < 0.05.

The addition of mucin directly stimulated *A. muciniphila* growth, and mucin is secreted by goblet cells *in vivo*. Thus, we examined the number of goblet cells in jejunum and colon in HFD and HFD-CAP mice at week 9. PAS staining was used to stain goblet cells (**Figures 6A,B**), and PAS-positive goblet cells per villus were counted. An average of 13 goblet cells was seen in HFD-CAP mice, which did not show a significant difference from that in HFD mice (**Figure 6C**).

To further explore the effect of CAP on goblet cells, sixteen 6-week C57BL/6J male mice were randomly divided into four groups (*n* = 4) and all were fed orally with CAP (0.4 mg in 200 μL PBS, see Materials and Methods). Gene expressions at four time points (0, 30, 60, and 90 min) in jejunum and proximal colon tissue samples were detected by qPCR. We tested the *Klf4* gene, a marker of goblet cells that regulated goblet cells’ differentiation. CAP did not directly increase *Klf4* mRNA expression in jejunum or colon within 90 min after the oral administration of CAP (**Figure 6D**).

We next examined the expression of the major goblet cell mucin production gene (*Muc2*) after CAP treatment. Mucin 2 mRNA expression was increased twofold at 30 min after CAP administration and lasted for 1.5 h in the jejunum (*P* < 0.05).

Last, we measured the mRNA expression of *Reg3g*, an antimicrobial protein that is predominantly expressed in the gastrointestinal tract (Wang et al., 2016). *Reg3g* has bactericidal activity and restricts bacterial colonization of mucosal surfaces. As is shown in **Figure 6D**, according the mean expression abundance values, *Reg3g* mRNA expression is steadily up-regulated in the colon after CAP administration and increased to about threefold at 90 min. However, the differences were not statistically significant.

## Discussion

Capsaicin is a worldwide consumed ingredient from chili peppers, and its anti-obesity function has been extensively studied through activation of TRPV1 channel (Yang et al., 2010; Ludy et al., 2012). Here, we found that the beneficial effects of CAP treatment on reducing body weight were associated with a modest modulation of gut microbiota. At the genus level, the proportion of *Bacteroides*, *Coprococcus*, *Prevotella*, and *Akkermansia* increased significantly. Recent studies indicated that obese individuals are associated with less *Bacteroides* (Qin et al., 2012; Johansson et al., 2013; Sonnenburg and Backhed, 2016; Wang and Jia, 2016), and the increased abundance of *Bacteroides* may be beneficial to the health. Genus *Coprococcus* was reported to be positively correlated with the GIP, (which is also known as glucose-dependent insulinotropic peptide) that induces insulin secretion (Thorens, 1995). Thus, the increase of *Coprococcus* might contribute to the improvement of glucose tolerance.

We observed an increase in the relative abundance of *A. muciniphila* at HFD-CAP mice. Recently, Baboota et al. (2014) used qPCR to detect the changes of seven gut microbes in HFD-CAP fed mice and found that the relative abundance of *A. muciniphila* increased. Interestingly, an increase of *Akkermansia* has also been reported in other microbiome studies. Anhê et al. (2015) found that a polyphenol-rich cranberry extract increased the relative abundance of *Akkermansia*, and the author claimed that the increase in *Akkermansia* population might prevent the production of the negative metabolic phenotype that is associated with obesity. Greer et al. (2016) found that *A. muciniphila* mediates negative effects of IFNγ on glucose metabolism. Kemperman et al. (2013) reported that complex polyphenols from black tea modulated the human gut microbial ecosystem, with an increase of *Akkermansia*. Roopchand et al. (2015) showed that dietary polyphenols promoted the growth of *A. muciniphila* and attenuate HFD-induced metabolic syndrome (Roopchand et al., 2015). Moreover, the antidiabetic drug metformin also has been found to modulate the gut microbiome and lined with an increased abundance of this bacterium (Shin et al., 2014). All these studies pointed out that a better health condition by dietary or pharmaceutical interventions is associated with increased *A. muciniphila*.

The probiotic effects of *A. muciniphila* have been studied extensively in animals and clinical studies, and the potential mechanisms were reported from cell biology studies (Derrien et al., 2011; Everard et al., 2013; Lukovac et al., 2014; Li et al., 2016). Therefore, we infer that the alteration of gut microbiota and increased *A. muciniphila* might contribute to the anti-obesity effects of CAP.

However, the mechanisms by which dietary or pharmaceutical interventions, including CAP, reshaped the gut microbiota and increased the relative abundance of *A. muciniphila* are still not unclear. Among all the current studies, the mechanism that directly stimulates the growth of *A. muciniphila in vivo* is lacking and a proposed mechanism is increased mucus production. We found that CAP inhibits the growth of *A. muciniphila* in high concentration and did not promote its growth at lower concentrations (**Figures 5A,B**). Since *A. muciniphila* is a mucin-degrading bacterium, *A. muciniphila* grows faster and plateaued at a higher cell density with the addition of mucin in the culture medium (**Figure 5C**). This result is consistent with previous reports (Derrien et al., 2004). Van den Abbeele et al. (2011) also showed that the abundance of *A. muciniphila* is positively correlated with the levels of mucins in the cecum. Therefore, we infer that CAP might stimulate mucin secretion in the gut to promote *A. muciniphila* growth.

Mucin is secreted by goblet cell, but we did not found a significant increase of goblet cell numbers in the colon after 9 weeks of CAP intervention, and CAP did not directly stimulate the expression of Kruppel-like factor (*Klf4*), the key factor that regulates goblet cell differentiation, in 90 min. However, an increased *Muc2* mRNA expression in the colon was observed after CAP treatment for 30 min. Interestingly, Karmouty-Quintana et al. (2007) showed that CAP administration induces mucus secretion in rat airways through the activation of sensory nerves. Therefore, we infer that CAP might stimulate mucin production and promote the growth of *A. muciniphila in vivo*, though the molecular mechanism needs further investigation.

More interestingly, we observed a steady increase of antimicrobial peptide *Reg3g* mRNA expression after CAP intervention. Antimicrobial peptides are produced and secreted by intestinal epithelial cells or Paneth cells and play a key role in maintaining the homeostasis in gut microbiota (Bevins and Salzman, 2011). *Reg3g* has bactericidal activity and keeps gut bacteria from the intestinal epithelial surface (Wang et al., 2016). Therefore, CAP might induce the antimicrobial defense to modulate the gut microbial composition. Further investigations are needed to better understand how CAP regulate gut microbiome and stimulate *A. muciniphila.*

Another potential explanation for the relative increase of *A. muciniphila* could be a decreased food intake in HFD-CAP fed mice. We observed a reduced food intake in HFD-CAP fed mice (**Supplementary Figure S1B**), which is consistent with previous studies (Cui and Himms-Hagen, 1992). Contrary to many other species, *A. muciniphila* can survive when host mucin is the only carbon and nitrogen source (Derrien et al., 2004, 2011), while most other microbes are dependent on food intake. Recently, Holmes et al. (2017) found that a reduced nutrient intake is associated with an increase in the abundance of *Akkermansia*. Thus, the decreased food intake might result in a higher relative abundance of *A. muciniphila* (**Figures 2E,F**).

## Conclusion

In summary, we found that CAP treatment reduces body weight and improves glucose homeostasis in HFD mice. This effect was associated with a modest modulation of gut microbiome. The changes in the gut microbiome and the increase in *Akkermansia* population might play a key role in this protective effect. Thus, we propose that CAP may prevent obesity through modulation of the gut microbiota.

## Availability Of Data And Material

The sequence data supporting the results of this article are available in the NCBI Sequence Read Archive under SRA accession number SRP082249.

## Ethics Statement

Animal experiments were carried out in strict accordance with the recommendations in the Guide for the Care and Use of Laboratory Animals and were approved by the Animal Care and Use Committee of the Third Military Medical University (Approval number: SYXC-2014-00203).

## Author Contributions

S. Le and FH conceived the study. WS, MS, XZ, HZ, YY, and S. Lu performed the experiments. WS, YT, and GL analyzed the sequence data. ML and WJ provided intellectual support. S. Le and WS wrote the paper. All authors read and approve the final manuscript for publication.

## Conflict of Interest Statement

The authors declare that the research was conducted in the absence of any commercial or financial relationships that could be construed as a potential conflict of interest.

## Footnotes

1 http://nbviewer.jupyter.org/github/biocore/qiime/blob/1.9.1/examples/ipynb/illumina_overview_tutorial.ipynb

2 http://picrust.github.io/picrust/tutorials/metagenome_prediction.html

3 http://picrust.github.io/picrust/tutorials/humann_tutorial.html

## Supplementary Material

FIGURE S1 **(A)** Weight gain of mice at week 9 (^∗^*P* < 0.05). **(B)** Average food intake (g/day/mice). **(C)** Area under the curve (AUC) of the oral glucose tolerance test (OGTT) (^∗^*P* < 0.05).

## Abbreviations

ATCC: American type culture collection
AUC: area under the curve
CAP: capsaicin
GIP: gastric inhibitory polypeptide
HFD: high-fat diet
KEGG: Kyoto Encyclopedia of Genes and Genomes
*Klf4*: Kruppel-like factor 4
*Muc2*: mucin 2 gene
NMDS: non-metric multi-dimensional scaling
OTU: operational taxonomic unit
PAS: Periodic Acid-Schiff
*Reg3g*: regenerating islet-derived 3 gamma
TRPV1: transient receptor potential vanilloid 1.

## References

[B1] Abubucker S. Segata N. Goll J. Schubert A. M. Izard J. Cantarel B. L. (2012). Metabolic reconstruction for metagenomic data and its application to the human microbiome. *PLoS Comput. Biol.* 8:e1002358 10.1371/journal.pcbi.1002358 PMC3374609 22719234

[B2] Anhê F. F. Roy D. Pilon G. Dudonné S. Matamoros S. Varin T. V. (2015). A polyphenol-rich cranberry extract protects from diet-induced obesity, insulin resistance and intestinal inflammation in association with increased *Akkermansia* spp. population in the gut microbiota of mice. *Gut* 64 872–883. 10.1136/gutjnl-2014-307142 25080446

[B3] Ayala J. E. Samuel V. T. Morton G. J. Obici S. Croniger C. M. Shulman G. I. (2010). Standard operating procedures for describing and performing metabolic tests of glucose homeostasis in mice. *Dis. Model Mech.* 3 525–534. 10.1242/dmm.006239 20713647 PMC2938392

[B4] Baboota R. K. Murtaza N. Jagtap S. Singh D. P. Karmase A. Kaur J. (2014). Capsaicin-induced transcriptional changes in hypothalamus and alterations in gut microbial count in high fat diet fed mice. *J. Nutr. Biochem.* 25 893–902. 10.1016/j.jnutbio.2014.04.004 24917046

[B5] Bevins C. L. Salzman N. H. (2011). Paneth cells, antimicrobial peptides and maintenance of intestinal homeostasis. *Nat. Rev. Microbiol.* 9 356–368. 10.1038/nrmicro2546 21423246

[B6] Bolger A. M. Lohse M. Usadel B. (2014). Trimmomatic: a flexible trimmer for Illumina sequence data. *Bioinformatics* 30 2114–2120. 10.1093/bioinformatics/btu170 24695404 PMC4103590

[B7] Caporaso J. G. Kuczynski J. Stombaugh J. Bittinger K. Bushman F. D. Costello E. K. (2010). QIIME allows analysis of high-throughput community sequencing data. *Nat. Methods* 7 335–336. 10.1038/nmeth.f.303 20383131 PMC3156573

[B8] Caporaso J. G. Lauber C. L. Walters W. A. Berg-Lyons D. Lozupone C. A. Turnbaugh P. J. (2011). Global patterns of 16S rRNA diversity at a depth of millions of sequences per sample. *Proc. Natl. Acad. Sci. U.S.A.* 108(Suppl. 1), 4516–4522. 10.1073/pnas.1000080107 20534432 PMC3063599

[B9] Collado M. C. Derrien M. Isolauri E. de Vos W. M. Salminen S. (2007). Intestinal integrity and *Akkermansia muciniphila*, a mucin-degrading member of the intestinal microbiota present in infants, adults, and the elderly. *Appl. Environ. Microbiol.* 73 7767–7770. 10.1128/AEM.01477-07 17933936 PMC2168041

[B10] Cui J. Himms-Hagen J. (1992). Long-term decrease in body fat and in brown adipose tissue in capsaicin-desensitized rats. *Am. J. Physiol.* 262(4 Pt 2), R568–R573. 1314515 10.1152/ajpregu.1992.262.4.R568

[B11] Dao M. C. Everard A. Aron-Wisnewsky J. Sokolovska N. Prifti E. Verger E. O. (2016). *Akkermansia muciniphila* and improved metabolic health during a dietary intervention in obesity: relationship with gut microbiome richness and ecology. *Gut* 65 426–436. 10.1136/gutjnl-2014-308778 26100928

[B12] Derbenev A. V. Zsombok A. (2016). Potential therapeutic value of TRPV1 and TRPA1 in diabetes mellitus and obesity. *Semin. Immunopathol.* 38 397–406. 10.1007/s00281-015-0529-x 26403087 PMC4808497

[B13] Derrien M. (2007). *Mucin Utilisation and Host Interactions of the Novel Intestinal Microbe Akkermansia muciniphila.* Ph.D. thesis, Wageningen University, Wageningen, The Netherlands.

[B14] Derrien M. Belzer C. de Vos W. M. (2016). *Akkermansia muciniphila* and its role in regulating host functions. *Microb. Pathog.* 10.1016/j.micpath.2016.02.005 [Epub ahead of print]. 26875998

[B15] Derrien M. Van Baarlen P. Hooiveld G. Norin E. Müller M. de Vos W. M. (2011). Modulation of mucosal immune response, tolerance, and proliferation in mice colonized by the mucin-degrader *Akkermansia muciniphila*. *Front. Microbiol.* 2:166 10.3389/fmicb.2011.00166 PMC3153965 21904534

[B16] Derrien M. Vaughan E. E. Plugge C. M. de Vos W. M. (2004). *Akkermansia muciniphila* gen. nov., sp. nov., a human intestinal mucin-degrading bacterium. *Int. J. Syst. Evol. Microbiol.* 54(Pt 5), 1469–1476. 10.1099/ijs.0.02873-0 15388697

[B17] DeSantis T. Z. Hugenholtz P. Larsen N. Rojas M. Brodie E. L. Keller K. (2006). Greengenes, a chimera-checked 16S rRNA gene database and workbench compatible with ARB. *Appl. Environ. Microbiol.* 72 5069–5072. 10.1128/AEM.03006-05 16820507 PMC1489311

[B18] Everard A. Belzer C. Geurts L. Ouwerkerk J. P. Druart C. Bindels L. B. (2013). Cross-talk between *Akkermansia muciniphila* and intestinal epithelium controls diet-induced obesity. *Proc. Natl. Acad. Sci. U.S.A.* 110 9066–9071. 10.1073/pnas.1219451110 23671105 PMC3670398

[B19] Gomez-Gallego C. Pohl S. Salminen S. De Vos W. M. Kneifel W. (2016). *Akkermansia muciniphila*: a novel functional microbe with probiotic properties. *Benef. Microbes* 7 571–584. 10.3920/BM2016.0009 27291403

[B20] Greer R. L. Dong X. Moraes A. C. Zielke R. A. Fernandes G. R. Peremyslova E. (2016). *Akkermansia muciniphila* mediates negative effects of IFNgamma on glucose metabolism. *Nat. Commun.* 7:13329 10.1038/ncomms13329 PMC5114536 27841267

[B21] Hachiya S. Kawabata F. Ohnuki K. Inoue N. Yoneda H. Yazawa S. (2007). Effects of CH-19 Sweet, a non-pungent cultivar of red pepper, on sympathetic nervous activity, body temperature, heart rate, and blood pressure in humans. *Biosci. Biotechnol. Biochem.* 71 671–676. 10.1271/bbb.60359 17341828

[B22] Holmes A. J. Chew Y. V. Colakoglu F. Cliff J. B. Klaassens E. Read M. N. (2017). Diet-microbiome interactions in health are controlled by intestinal nitrogen source constraints. *Cell Metab.* 25 140–151. 10.1016/j.cmet.2016.10.021 27889387

[B23] Johansson M. E. V. Sjövall H. Hansson G. C. (2013). The gastrointestinal mucus system in health and disease. *Nat. Rev. Gastroenterol. Hepatol.* 10 352–361. 10.1038/nrgastro.2013.35 23478383 PMC3758667

[B24] Karmouty-Quintana H. Cannet C. Sugar R. Fozard J. R. Page C. P. Beckmann N. (2007). Capsaicin-induced mucus secretion in rat airways assessed in vivo and non-invasively by magnetic resonance imaging. *Br. J. Pharmacol.* 150 1022–1030. 10.1038/sj.bjp.0707168 17351665 PMC2013907

[B25] Kawabata F. Inoue N. Masamoto Y. Matsumura S. Kimura W. Kadowaki M. (2009). Non-pungent capsaicin analogs (capsinoids) increase metabolic rate and enhance thermogenesis via gastrointestinal TRPV1 in mice. *Biosci. Biotechnol. Biochem.* 73 2690–2697. 10.1271/bbb.90555 19966466

[B26] Kawabata F. Inoue N. Yazawa S. Kawada T. Inoue K. Fushiki T. (2006). Effects of CH-19 sweet, a non-pungent cultivar of red pepper, in decreasing the body weight and suppressing body fat accumulation by sympathetic nerve activation in humans. *Biosci. Biotechnol. Biochem.* 70 2824–2835. 10.1271/bbb.60206 17151481

[B27] Kemperman R. A. Gross G. Mondot S. Possemiers S. Marzorati M. Van de Wiele T. (2013). Impact of polyphenols from black tea and red wine/grape juice on a gut model microbiome. *Food Res. Int.* 53 659–669. 10.1016/j.foodres.2013.01.034

[B28] Langille M. G. Zaneveld J. Caporaso J. G. McDonald D. Knights D. Reyes J. A. (2013). Predictive functional profiling of microbial communities using 16S rRNA marker gene sequences. *Nat. Biotechnol.* 31 814–821. 10.1038/nbt.2676 23975157 PMC3819121

[B29] Lee E. Jung D. Y. Kim J. H. Patel P. R. Hu X. Lee Y. (2015). Transient receptor potential vanilloid type-1 channel regulates diet-induced obesity, insulin resistance, and leptin resistance. *FASEB J.* 29 3182–3192. 10.1096/fj.14-268300 25888600 PMC4511197

[B30] Li J. Lin S. Vanhoutte P. M. Woo C. W. Xu A. (2016). *Akkermansia muciniphila* protects against atherosclerosis by preventing metabolic endotoxemia-induced inflammation in Apoe-/- mice. *Circulation* 133 2434–2446. 10.1161/CIRCULATIONAHA.115.019645 27143680

[B31] Ludy M. J. Moore G. E. Mattes R. D. (2012). The effects of capsaicin and capsiate on energy balance: critical review and meta-analyses of studies in humans. *Chem. Senses* 37 103–121. 10.1093/chemse/bjr100 22038945 PMC3257466

[B32] Lukovac S. Belzer C. Pellis L. Keijser B. J. de Vos W. M. Montijn R. C. (2014). Differential modulation by *Akkermansia muciniphila* and *Faecalibacterium prausnitzii* of host peripheral lipid metabolism and histone acetylation in mouse gut organoids. *MBio* 5:e1438-14 10.1128/mBio.01438-14 PMC4145684 25118238

[B33] Luo Z. Ma L. Zhao Z. He H. Yang D. Feng X. (2012). TRPV1 activation improves exercise endurance and energy metabolism through PGC-1α upregulation in mice. *Cell Res.* 22 551–564. 10.1038/cr.2011.205 22184011 PMC3292293

[B34] Magoc T. Salzberg S. L. (2011). FLASH: fast length adjustment of short reads to improve genome assemblies. *Bioinformatics* 27 2957–2963. 10.1093/bioinformatics/btr507 21903629 PMC3198573

[B35] Ogden C. L. Yanovski S. Z. Carroll M. D. Flegal K. M. (2007). The epidemiology of obesity. *Gastroenterology* 132 2087–2102. 10.1053/j.gastro.2007.03.052 17498505

[B36] Parks D. H. Tyson G. W. Hugenholtz P. Beiko R. G. (2014). STAMP: statistical analysis of taxonomic and functional profiles. *Bioinformatics* 30 3123–3124. 10.1093/bioinformatics/btu494 25061070 PMC4609014

[B37] Qin J. Li Y. Cai Z. Li S. Zhu J. Zhang F. (2012). A metagenome-wide association study of gut microbiota in type 2 diabetes. *Nature* 490 55–60. 10.1038/nature11450 23023125

[B38] Roopchand D. E. Carmody R. N. Kuhn P. Moskal K. Turnbaugh P. J. Raskin I. (2015). Dietary polyphenols promote growth of the gut bacterium *Akkermansia muciniphila* and attenuate high fat diet-induced metabolic syndrome. *Diabetes* 64 2847–2858. 10.2337/db14-1916 25845659 PMC4512228

[B39] Segata N. Izard J. Waldron L. Gevers D. Miropolsky L. Garrett W. S. (2011). Metagenomic biomarker discovery and explanation. *Genome Biol.* 12:R60 10.1186/gb-2011-12-6-r60 PMC3218848 21702898

[B40] Shin N.-R. Lee J.-C. Lee H.-Y. Kim M.-S. Whon T. W. Lee M.-S. (2014). An increase in the *Akkermansia* spp. population induced by metformin treatment improves glucose homeostasis in diet-induced obese mice. *Gut* 63 727–735. 10.1136/gutjnl-2012-303839 23804561

[B41] Sonnenburg J. L. Backhed F. (2016). Diet-microbiota interactions as moderators of human metabolism. *Nature* 535 56–64. 10.1038/nature18846 27383980 PMC5991619

[B42] Thorens B. (1995). Glucagon-like peptide-1 and control of insulin secretion. *Diabete Metab.* 21 311–318. 8586147

[B43] Van den Abbeele P. Gerard P. Rabot S. Bruneau A. El Aidy S. Derrien M. (2011). Arabinoxylans and inulin differentially modulate the mucosal and luminal gut microbiota and mucin-degradation in humanized rats. *Environ. Microbiol.* 13 2667–2680. 10.1111/j.1462-2920.2011.02533.x 21883787

[B44] van Passel M. W. J. Kant R. Zoetendal E. G. Plugge C. M. Derrien M. Malfatti S. A. (2011). The genome of *Akkermansia muciniphila*, a dedicated intestinal mucin degrader, and its use in exploring intestinal metagenomes. *PLoS ONE* 6:e16876 10.1371/journal.pone.0016876 PMC3048395 21390229

[B45] Wang J. Jia H. (2016). Metagenome-wide association studies: fine-mining the microbiome. *Nat. Rev. Microbiol.* 14 508–522. 10.1038/nrmicro.2016.83 27396567

[B46] Wang L. Fouts D. E. Starkel P. Hartmann P. Chen P. Llorente C. (2016). Intestinal REG3 lectins protect against alcoholic steatohepatitis by reducing mucosa-associated microbiota and preventing bacterial translocation. *Cell Host Microbe* 19 227–239. 10.1016/j.chom.2016.01.003 26867181 PMC4786170

[B47] Yang D. Luo Z. Ma S. Wong W. T. Ma L. Zhong J. (2010). Activation of TRPV1 by dietary capsaicin improves endothelium-dependent vasorelaxation and prevents hypertension. *Cell Metab.* 12 130–141. 10.1016/j.cmet.2010.05.015 20674858 PMC3906919

