# Supplementary figures and images for: Anti-obesity Effect of Capsaicin in Mice Fed with High-Fat Diet Is Associated with an Increase in Population of the Gut Bacterium Akkermansia muciniphila
Source: Front Microbiol. 2017 Feb 23;8:272. doi: 10.3389/fmicb.2017.00272 (PMC5322252; doi:10.3389/fmicb.2017.00272)

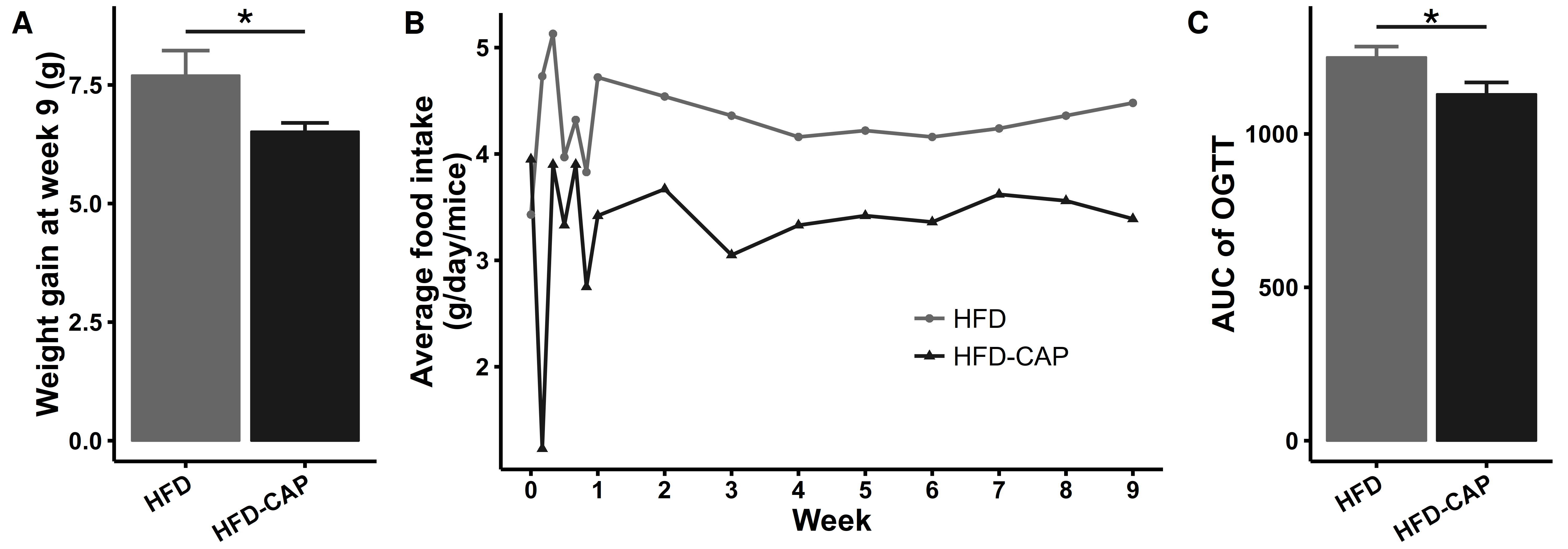

Supplement: FIGURE S1 — (A) Weight gain of mice at week 9 (∗P < 0.05). (B) Average food intake (g/day/mice). (C) Area under the curve (AUC) of the oral glucose tolerance test (OGTT) (∗P < 0.05). [file Image_1.TIFF]
